# In Search of Breast Cancer Culprits: Suspecting the Suspected and the Unsuspected

**Published:** 2008-01-15

**Authors:** Goberdhan P. Dimri

**Affiliations:** Department of Medicine, ENH Research Institute, Robert H. Lurie Comprehensive Cancer Center, Feinberg School of Medicine, Northwestern University, Evanston, IL 60201

## Abstract

I would like to welcome breast cancer research community to the first editorial of our newest journal “Breast Cancer: Basic and Clinical Research”. In pursuit of breast cancer culprits, we have come a long way since the early 90’s when the first breast cancer susceptibility gene *BRCA1* was mapped and cloned. In the past few years, several new loci associated with the various degree of breast cancer risk have been identified using “Candidate Gene Association Study (CGAS) and Genome-Wide Association Study (GWAS)” approaches. This editorial is meant to quickly glance over recent findings of these population-based association studies.

## Introduction

Breast cancer is one of the most terrifying diseases that human civilization has ever known. Legend has it that powerful queen Atossa of the ancient Babylon had breast cancer, and the specific mention of breast cancer can be found in “Hippocratic Corpus” written by Hippocrates and his peers dating back to fourth and fifth centuries B.C. (Karpozilos and Pavlidis, 2004). Throughout our past and present civilization, we are reminded that several notable figures and ordinary citizens have suffered or are suffering from this dreadful disease. Although the life-time risk of developing breast cancer may vary in different geographic regions of the world, nobody is immune to developing breast cancer. In the United States of America and most western countries, the life-time risk of developing breast cancer in women is close to 1 in 8. The most intriguing question is- what determines this risk?

It is well known that early onset breast cancer tends to cluster in families and usually first degree relatives of affected individuals have twofold higher risk of developing breast cancer (2001). This increased risk is independent of lifestyle and environmental factors, and thought to be due to genetic susceptibility of individuals to develop breast cancer (Lichtenstein et al. 2000). The early onset breast cancer, which tends to cluster in families, is also known as familial breast cancer. Overall, 20%–25% of familial breast cancer is attributed to high penetrance genes *BRCA1*, *BRCA2*, *TP53*, and *PTEN* (Easton, 1999).

The high penetrance genes were identified using family-based linkage studies. These studies also identified additional breast cancer susceptibility genes. In all, these family-based linkage studies were instrumental in identifying ten important genes- *BRCA1, BRCA2, TP53, PTEN, CHEK2, ATM, NBS1, RAD50, BRIP1,* and *PALB2* for inherited breast cancer (Walsh and King, 2007). These ten genes, which are critical for genome integrity account for roughly 50% of familial breast cancer (Walsh and King, 2007). Despite intense efforts, linkage studies have failed to identify additional breast cancer susceptibility genes for familial breast cancer.

The late onset breast cancer, which is primarily sporadic in nature, is by far the most prevalent. In sporadic breast cancer, “the 10 genes for inherited breast cancer” have very minimal role. Hence, the 50% of the familial or early onset cases and majority of late onset cases of breast cancer must involve low to medium penetrance genes. The linkage studies lack the power to detect alleles responsible for low to moderate risk of developing breast cancer. Such alleles are now being identified using population-based gene-association studies. These studies take advantage of thousands of known single-nucleotide polymorphisms (SNPs) present in the human genome. The earlier studies focused on candidate gene approach and looked for SNPs in limited number of genes and their possible association with breast cancer. More recent studies known as Genome-Wide Association Studies (GWASs) are taking advantage of unbiased scan of the whole genome for SNPs associated with breast cancer risk.

## The Usual Suspects: Candidate Gene Association Studies

Several breast cancer research groups have studied the association of breast cancer risk with common variants (SNPs) of candidate genes. More comprehensive CGASs have been carried out by SEARCH breast cancer study group and the “Breast Cancer Association Consortium” (BCAC). Results of CGASs have been mixed and very confusing. Some of the association studies may not be directly comparable because they used different population groups, while in other studies, the sample size may not be sufficiently large enough.

Starting with *HER-2*, in some studies a common variant *HER-2* V655I was reported to be associated with breast cancer (Xie et al. 2000), however other studies found no such association (Benusiglio et al. 2006; Einarsdottir et al. 2006). Human *CYP19* gene, which encodes aromatase cytochrome P450 is another plausible candidate gene where some studies have suggested association of common variants with significant breast cancer risk (Haiman et al. 2003; Ralph et al. 2007), while other studies suggested no association of any SNPs in *CYP19* with breast cancer risk (Healey et al. 2000). Recently, Ralph et al. suggested possible age-specific association of certain SNPs present in genes encoding steroid hormone pathway (Ralph et al. 2007). Specifically, it was reported that cytosine/cytosine homozygous genotype of cytochrome P450 XIB2 (*CYP11B2*) was associated with reduced breast cancer risk at younger age, but increased risk at older age (Ralph et al. 2007), and homozygous cytosine-guanine (CG/CG) genotype of uridine phosphorylase glycosyltransferase 1A7 (*UGT1A7*) was associated with increased breast cancer risk at younger ages but decreased risk at older ages (Ralph et al. 2007).

By analyzing 4,474 breast cancer cases and 4,560 controls from SEARCH collection (United Kingdom), Baynes et al. reported that in contrast to rare variants, the common variants in the *ATM, BRCA1, BRCA2, CHEK2* and *TP53* are unlikely to increase the breast cancer risk (Baynes et al. 2007). A study be Onay et al. also concluded that 19 individual commonly occurring SNPs associated with 18 key cancer genes *XPD, PTEN, GADD45, p27, ESR1, CYP17, GSTM3, MTHFR, IL1a, IL10, IL13, TNFa, G-CSF, CCND1, COMT, BARD1, GSTP1* and *MMP1* did not contribute to breast cancer risk (Onay et al. 2006). A very recent BCAC study also did not find an association of *MDM2* SNP309 and *TP53* R72P SNP with breast cancer risk (Schmidt et al. 2007).

On the other hand, common variants of few genes, which were suspected to play a role in breast cancer did turned out to have weak association with breast cancer risk. For example, BCAC reported that common coding variants *CASP8* D302H in the gene encoding Caspase 8, and *TGFB1* L10P in the gene encoding transforming growth factor-β (TGFβ), in one allele (heterozygote) were associated with significant risk to invasive breast cancer (Cox et al. 2007). In an earlier study, *CASP8* D302H variant was reported to be associated with reduced breast cancer risk in a dose-dependent manner; it provided better protection against breast cancer in the homozygous condition (MacPherson et al. 2004).

BCAC also analyzed data from 12 studies for 16 SNPs in various candidate genes and concluded that only 5 SNPS (*CASP*8 D302H, *IGFBP3*-202 *c* > *a*, *PGR*V660L, *SOD2* V16A, and *TGFB1* L10P) were associated with breast cancer, but the statistical significance of the association was only borderline (Breast Cancer Association, 2006). The remaining 11 SNPs in other candidate genes showed no significant association with breast cancer risk (Breast Cancer Association, 2006). Another recent study (SEARCH investigators), which analyzed association between common variants found in 120 candidate genes and breast cancer concluded that a proportion of SNPs in candidate genes in the cell-cycle control pathway, genes involved in steroid hormone metabolism and signaling were weakly associated with breast cancer risk but large sample-sizes from multicentre collaboration is needed to identify SNPs that are associated with definitive breast cancer risk (Pharoah et al. 2007). Some borderline significance of SNPs in few selected antioxidant defense genes (for example *CAT g*27168*a, TXN t*2715*c, TXNRD2* A66S and *TXNRD2 g*23524*a*) and epigenetic genes (for example *DNMT3b*-*c*31721*t*) with breast cancer risk has been reported but these observations need to be confirmed in larger epidemiological studies (Cebrian et al. 2006a; Cebrian et al. 2006b).

## Not so Usual Suspects: Genome-Wide Association Studies

As discussed above, the candidate gene approach studies to identify breast cancer risk has not been very successful. With the advent of rapid SNP screening technologies and completion of “Hap Map”, it is now possible to rapidly scan the genome of several thousand individuals to find association of SNPs with a particular disease. Recently, four such GWASs have been conducted to identify novel breast cancer susceptibility loci (Easton et al. 2007; Hunter et al. 2007; Murabito et al. 2007; Stacey et al. 2007).

Stacey et al. genotyped 4,554 breast cancer patients and 17,577 controls using the Illumina Hap300 platform and reported that individuals of European descent with homozygous allele A of rs13387042 SNP on chromosome 2q35 have an estimated 1.44 fold higher risk of estrogen receptor-positive (ER-positive) breast cancer compared to noncarriers, while homozygous allele T of rs3803662 on chromosome 16q12 was associated with 1.64 fold risk of ER-positive breast cancer (Stacey et al. 2007). Among other ethnicities, both variants were only marginally significant; in fact T-rs3803662 allele was protective in African Americans (Stacey et al. 2007). Functional significance of both these SNPs is not clear, although rs3803662 is near the 5’ end of *TNRC9*, a gene implicated in bone metastasis of breast cancer cells. Remarkably, significant breast cancer association of rs3803662 SNP near the 5’ end of *TNRC9* was also reported in an independent study (Easton et al. 2007).

The discovery of association of SNPs in intron 2 of *FGFR2*, which encode fibroblast growth factor receptor 2, with breast cancer risk was also reported in two independent GWASs (Easton et al. 2007; Hunter et al. 2007). In the first study, which also tagged rs3803662, GWAS was carried out using a two-stage analysis of 4,398 breast cancer cases and 4,316 controls. At second stage, authors found significant association of 1,792 SNPs with breast cancer risk, but chose to study 30 SNPs with highest level of significance for subsequent confirmation in 21,860 cases and 22,578 controls chosen from 22 studies (Easton et al. 2007). The following SNPs showed the most significant and consistent evidence of association- rs2981582 (*FGFR2*), and rs12443621, rs8051542 and rs3803662 (*TNRC9*), rs889312 (*MAP3K1*), rs13281615 (8q) and rs3817198 (*LSP1*) (Easton et al. 2007). Although *FGFR*, *TNRC9*, *MAP3K1 and LSP1* are plausible breast cancer culprits, the functional significance of SNPs in these genes remain unclear at this point (Easton et al. 2007).

In the second GWAS, Hunter et al. genotyped 528,173 SNPs in 1,145 postmenopausal women of European descent with invasive breast cancer and 1,142 controls (Hunter et al. 2007). The GWAS identified four SNPs (rs1219648, rs2420946, rs11200014 and rs2981579) in intron 2 of *FGFR2*, which showed significant association with breast cancer (Hunter et al. 2007). The association was confirmed using three additional studies using 1,776 cases and 2,072 controls (Hunter et al. 2007). Again, although *FGFR2* is a plausible breast cancer gene, the functional significance of these common variants in *FGFR2* loci is not clear.

Another GWAS was conducted by Murabito et al. using study subjects from NHLBI’s Framingham Heart Study (Murabito et al. 2007). The study involved 1,335 participants, including 58 women with breast cancer and 59 men with prostate cancer (Murabito et al. 2007). Possibly, because of limited size of the population, authors did not find significant association of any SNP with breast or prostate cancer risk. Although in the same study, using candidate gene approach, authors reported significant association of two SNPs (rs9325782 and rs2410373) in *MSRI* gene with prostate cancer, and three SNPs (rs905883, rs7564590 and rs7558615) in *ERBB4* with breast cancer (Murabito et al. 2007).

## Conclusion: Devil is Hiding in the Genome

With the rapid advent of genotyping technologies, we have entered an exciting era of genome-based discoveries for human diseases. CGAS and GWAS clearly have the power to identify common variants that are associated with low susceptibility loci for a particular disease. At present, due to continued drop in genotyping costs, GWAS appears to be a better approach than CGAS. However, a great degree of caution is needed in the correct interpretation of such studies. There are several issues which need to be addressed in each GWAS; the caveats range from sample size to genotyping quality controls to successful replication of results. Several of these points are discussed in NCI-NHGRI (National Cancer Institute-National Human Genome Research Institute) working group recommendations on replicating GWAS results (Chanock et al. 2007).

The next legitimate question is- what is the overall risk of a particular disease associated with these so called common variants? Although statistically significant, the effect of individual SNP is generally very small in terms of increasing breast cancer risk. Such risk is usually close to 1.2 to 1.5. However, the overall effect of combinations of SNPs on breast cancer risk may be substantial. This overall effect, which is also termed as polygenic effect (of SNPs) may be additive or synergistic and may account for most of the genetic risk associated with developing breast cancer. Although, only a handful of these common variants have been identified so far, the presumption is that there are many more of these, and they may genetically interact.

For now, the breast cancer community will wait for the identification of all of the common variants in the genome, which would be associated with breast cancer, and then all of us- clinical and basic scientists, and other stake holders will debate what is in the best interest of naïve general public. Should we prepare for the genetic counseling of would be breast cancer patients even though the overall risk factor may still be below 1.5 to 2.0? In summary, each GWAS starts with an assumption that the evildoers are in the genome and that they most probably conspire together to increase the risk of developing a particular disease such as breast cancer. On an optimistic note, the gene hunters or SNP hunters to be more accurate, are busy hunting these evildoers, wherever they may be- in the introns, exons or the regulatory regions in the genome.
